# Mannose-6-phosphate facilitates early peripheral nerve regeneration in *thy-1-YFP-H* mice

**DOI:** 10.1016/j.neuroscience.2014.08.034

**Published:** 2014-10-24

**Authors:** A.J. Harding, C.R. Christmas, M.W.J. Ferguson, A.R. Loescher, P.P. Robinson, F.M. Boissonade

**Affiliations:** a Unit of Oral and Maxillofacial Medicine and Surgery, School of Clinical Dentistry, University of Sheffield, Claremont Crescent, Sheffield S10 2TA, UK; b Renovo Group plc, Core Technology Facility, 48 Grafton Street, Manchester M13 9XX, UK

**Keywords:** ANOVA, analysis of variance, IL, interleukin, LAP, latency-associated peptide, LTBP, latent TGF-β-binding protein, M6P, mannose-6-phosphate, TGF, transforming growth factor, WT, wild-type, YFP, yellow fluorescent protein, nerve regeneration, nerve repair, mannose-6-phosphate, *thy-1-YFP-H*, scarring

## Abstract

The formation of scar tissue following nerve injury has been shown to adversely affect nerve regeneration and evidence suggests that mannose-6-phosphate (M6P), a potential scar reducing agent that affects transforming growth factor (TGF)-β activation, may enhance nerve regeneration. In this study we utilized *thy-1-YFP-H* mice – a transgenic strain expressing yellow fluorescent protein (YFP) within a subset of axons – to enable visual analysis of axons regenerating through a nerve graft. Using this strain of mouse we have developed analysis techniques to visualize and quantify regeneration of individual axons across the injury site following the application of either M6P or vehicle to the site of nerve injury.

No significant differences were found in the proportion of axons regenerating through the graft between M6P- and vehicle-treated grafts at any point along the graft length. Maximal sprouting occurred at 1.0 mm from the proximal graft ending in both groups. The maximum change in sprouting levels for both treatment groups occurred between the graft start and 0.5-mm interval for both treatment groups. The difference between repair groups was significant at this point with a greater increase seen in the vehicle group than the M6P group. The average length of axons regenerating across the initial graft entry was significantly shorter in M6P- than in vehicle-treated grafts, indicating that they encountered less impedance.

Application of M6P appears to reduce the disruption of regenerating axons and may therefore facilitate quicker recovery; this is likely to result from altered scar tissue formation in M6P grafts in the early stages of recovery. This study also establishes the usefulness of our methods of analysis using the *thy-1-YFP-H* mouse strain to visualize and quantify regeneration at the level of the individual axon.

## Highlights

• We have visualized and quantified nerve regeneration at an axonal level.
• Axons cross the repair site more directly following mannose-6-phosphate treatment.
• Mannose-6-phosphate alters axon sprouting just distal to axon entry into the graft.
• Mannose-6-phosphate may enable more favorable collagen fibril alignment.
• Our data add further evidence that mannose-6-phosphate improves nerve regeneration.

## Introduction

Peripheral nerve injuries are a common occurrence worldwide with over 300,000 cases annually in Europe alone (Ciardelli and Chiono, 2006). Most occur through domestic, workplace and traffic accidents but a significant number are caused by surgical procedures (Ciaramitaro et al., 2010). In one study Kretschmer et al. (2001) reported that 17.4% of traumatic nerve lesions treated over a 9-year period at one nerve-injury centre were iatrogenic in origin.

It has been well documented that peripheral nerves are able to undergo regeneration following injury; however functional recovery following surgery is unpredictable and is rarely complete (Robinson et al., 2000). The formation of intra-neural scarring following peripheral nerve injury has long been considered to adversely affect functional recovery in peripheral nerves, with Bora (1967) noting a negative correlation between the quantity of fibrous tissue at a nerve repair site and the overall level of functional recovery. More recently Atkins et al. (2006b) provided ‘proof of concept’ in a study that demonstrated that reducing intra-neural scarring improved nerve regeneration. In Atkins’ study, regeneration of the sciatic nerve in wild-type (WT) mice was compared with that in two transgenic strains – one with an increased propensity for scarring (interleukin (IL)-4/IL-10 null mice) and the other with a decreased propensity for scarring (M6PR/IGF2 null mice) – with the results demonstrating that reducing the level of scarring at the injury site could improve nerve regeneration.

Alteration of the ratios of transforming growth factor (TGF)-β1, -β2 and -β3 at the site of injury has been shown to affect the level of scar formation in dermal wounds (Shah et al., 1994, Shah et al., 1995), with neutralisation of TGF-β1 and -β2 using antibodies, and exogenous addition of TGF-β3 reducing scarring in rat models. Neutralisation of TGF-β1 and -β2 at the site of a nerve injury has also been shown to significantly reduce the level of scarring (Atkins et al., 2006a). However the effect upon nerve regeneration is less clear, with Atkins et al. (2006a) reporting no improvement in regeneration and Davison et al. (1999), who neutralised only TGF-β1, reporting a significant improvement.

As TGF-β1 is linked to promoting the transition of Schwann cells into their more active, proliferating, non-myelinating phenotype following nerve injury (Einheber et al., 1995), the complete removal of TGF-β1 from the injury site may negatively affect some aspects of regeneration. An alternative approach to using TGF-β antibodies to reduce intra-neural scarring is the use of mannose-6-phosphate (M6P); which has been shown to reduce scarring in dermal wounds (McCallion and Ferguson, 1996). TGF-β is secreted from cells in a latent form bound to a latency-associated peptide (LAP) and latent TGF-β-binding protein (LTBP), and only becomes active once LAP and LTBP are removed; M6P occupies receptors used to trigger the removal of LAP and LTBP and as such reduces local TGF-β activation (McCallion and Ferguson, 1996).

A previous study carried out in our laboratory, looking at the effects of M6P on nerve regeneration, found that the application of M6P to a nerve repair did produce a significant improvement in nerve regeneration compared to controls at 6 weeks post repair (Ngeow et al., 2011b). However, this was not accompanied by a reduction in scarring, and at 12 weeks post repair there was no significant difference between M6P-treated and control repairs. The analytical methods used by the authors to assess nerve regeneration (walking gait analysis and electrophysiology) are well-established methods; however, they do have limitations in that they only indicate whether a particular treatment has improved regeneration and give no indication of why some axons fail to cross the repair site.

In this study we utilized *thy-1-YFP-H* mice – a transgenic strain expressing yellow fluorescent protein (YFP) within a subset of axons (Feng et al., 2000) – to enable visual analysis of axons regenerating through a nerve graft. This strain of mouse has been used in previous studies of peripheral nerve regeneration (English et al., 2005, English et al., 2007, Groves et al., 2005) as it allows the path of individual axons to be traced across the injury site. Using this strain of mouse we have developed new methods of analysis to visualize and quantify regeneration of individual axons following the application of either M6P or vehicle to the site of nerve injury.

## Experimental procedures

### Surgical procedure

Fifty-three mice aged between 9 and 12 weeks (at the time of the initial surgery) were used in this study: 33 *thy-1-YFP-H* (YFP+) mice and 20 C57B/6J (WT) mice. The WT mice used were littermates of the YFP+ mice. The experiments were carried out under appropriate UK Home Office approval, in accordance with the Animals (Scientific Procedures) Act 1986. The experimental model involved unilateral repair of the common fibular nerve with a nerve graft treated with either M6P (600 mM) or vehicle (phosphate-buffered saline). The experimental groups were: M6P-treated grafts (*n* = 10 YFP+ mice and *n* = 10 WT mice), vehicle-treated grafts (*n* = 10 YFP+ mice and 10 WT mice), and uninjured controls (*n* = 10 YFP+ mice). A further group (*n* = 3 YFP+ mice) was used to assess the level of residual fluorescence in the nerve distal to the injury site when graft repair was not carried out.

Under general anesthesia (2–3% isoflurane; Abbott Laboratories, Maidenhead, UK), the common fibular nerve of a WT mouse was exposed and freed from the surrounding tissue. A section of nerve (5 mm minimum length) was then removed and placed in a numbered vial containing either M6P or vehicle (randomized with the investigator blind to the contents). The nerve tissue remained immersed in the solution for 30 min. Following the removal of the nerve, WT animals were euthanized by cervical dislocation while under deep anesthesia.

During the period of immersion a YFP+ littermate was prepared to receive the graft. Under anesthesia (2–3% isoflurane), the right common fibular nerve was exposed and carefully freed from the surrounding tissue. A 5-mm silicone trough was then inserted beneath the nerve, and the nerve was sectioned. A gap of 2.5–3 mm was created between the proximal and distal ends by careful trimming and the treated WT graft (as described above) was trimmed to fit the gap. The graft and nerve ends were then aligned and the graft was secured in place with fibrin glue, consisting of fibrinogen (10 mg/ml; Sigma–Aldrich, Gillingham, Dorset, UK) and thrombin (40 units/ml; Sigma–Aldrich, UK) in a 1:1 ratio, and allowed to set for 5 min. Once the repair was secure the silicone trough was carefully removed and the wound closed. A single dose of analgesic was administered subcutaneously (0.01 ml buprenorphine hydrochloride 0.3 mg/ml; Vetergesic®, Alstoe Animal Health, Sheriff Hutton, UK) and the mice were then allowed to recover for 2 weeks.

Following the recovery period, mice were re-anaesthetized (fentanyl/fluanisone, 0.8 ml/kg [Hypnorm, Janssen Animal Health, High Wycombe, UK] and midazolam, 4 mg/kg [Hypnovel, Roche Products Ltd, Welwyn Garden City, UK]; ip) and the common fibular nerve exposed and freed from surrounding tissue. The skin was sutured to a brass ring to form a pool, which was filled with 4% paraformaldehyde for 30 min to fix the nerve. Following fixation the nerve was excised and mounted on a microscope slide using Vectashield®, and the mouse culled under deep anesthesia by cervical dislocation. The uninjured control nerves were also obtained using this procedure.

To assess residual fluorescence in the portion of the nerve distal to the injury, the common fibular nerve in three YFP+ mice was exposed and sectioned as described above. Following sectioning the proximal end was tied off with a silk suture to prevent regeneration of axons. Following a 2-week recovery period the proximal and distal portions of the injured nerves were fixed and excised as described above.

### Image acquisition and processing

**Fig. 1:**
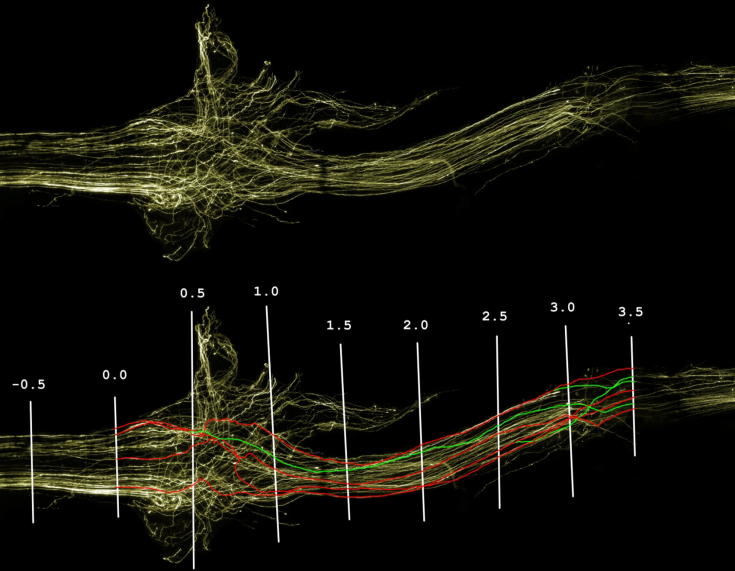
Reconstructed nerve image. A typical reconstructed nerve image (top) and the same image with 0.5-mm intervals marked and sample axons traced from the 3.5-mm interval to the graft start (green lines indicating duplicate branches of the axons traced in red).

Images of nerves were acquired with fluorescent microscopy (Zeiss Axioplan 2 imaging microscope with QImaging Retiga 1300R camera) using the Optigrid Structured Illumination system and Image Pro-Plus software (version 5; Media Cybernetics, Bethesda, MD, USA). Images were acquired with a 10× objective in sequential steps in the *z*-axis. The composite images shown in the figures were created from stacks of thirty 10-μm-thick sections throughout the entire thickness of the nerve. To obtain images of the full length of the nerve, stacks were acquired from multiple adjoining microscope fields and the composite images were joined using Adobe Photoshop (Fig. 1).

Minimal image processing with Adobe Photoshop was applied to the joined images used for analysis; this consisted of adjusting brightness/contrast and image exposure in order to improve axon clarity.

### Image analysis

All analysis was carried out with the assessor blind to the treatment received by each nerve graft.

The first point at which abnormal morphology could be determined by observation was marked by a perpendicular line. Using this line as a reference, further lines were drawn at 0.5-mm intervals (Fig. 1); one 0.5 mm proximal to the reference line and the remainder up to 3.5 mm distal to the reference line. The analysis site proximal to the reference line was referred to as the pre-graft interval with the other sites referred to as their distance in mm from the reference line (0.5 mm, 1.0 mm, etc.). For uninjured controls a perpendicular line was marked 0.5 mm from the proximal end of the nerve section and further lines were marked at 0.5-mm intervals distal to the first.

A sprouting index was calculated for each interval by counting the number of axons at the selected interval and dividing by the number of axons at the pre-graft interval. This gives an indication of whether the number of axons at a specific point has increased or decreased. Additionally, at each interval the change in sprouting index compared to the previous interval was calculated.

In order to calculate the number of individual axons from the graft start that successfully regenerated through the length of the graft, axons at the 3.5-mm interval were traced back along their length to the graft start (Fig. 1). A total of 75% of the axons present at the 3.5-mm interval were traced. This was considered to be the largest percentage of axons that could be accurately traced. The trace finished either when the axon reached the graft start point or joined up with a branch point of a previously traced axon. It was decided to trace axons in this manner as tracing axons from the graft start toward the 3.5-mm interval would involve frequent subjective decisions to be made with regard to which axon branch to follow and would affect repeatability.

**Fig. 2:**
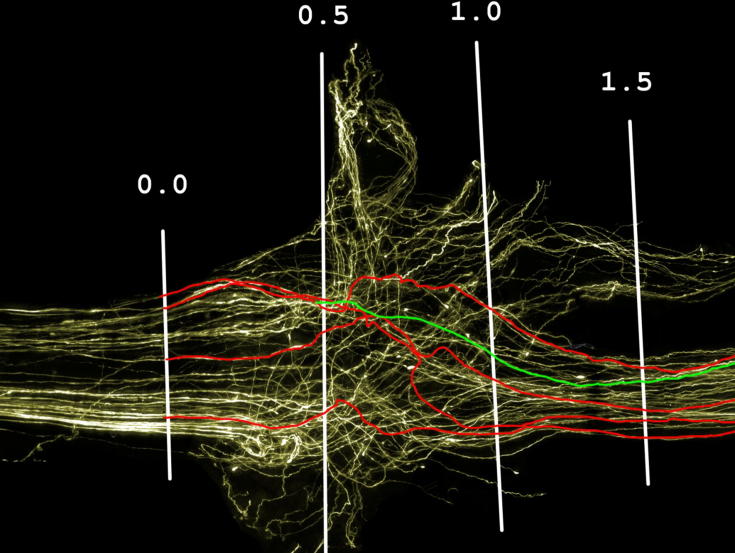
Enlarged view of the initial injury site. The length of traced axons was measured between the graft start and the 1.5-mm interval and compared to the direct measurement between the two points.

Finally, the shortest direct route between the 0.0-mm and 1.5-mm intervals was measured along with the length of the traced axons over the same distance (ImageJ, National Institutes of Health, Bethesda, MD, USA) (Fig. 2); the 1.5-mm interval was the first interval beyond the graft entry disruption in the majority of repairs. This was used to calculate the average percentage of axon length above the minimum possible per graft to provide an indication of the level of axonal disruption as they entered the graft. Theoretically, axons with a fairly clear, unobstructed path to follow would be shorter than those having to make their way around obstructions such as scar tissue.

### Statistical analysis

Statistical comparisons between groups were made using GraphPad Prism (version 5.00 for Windows; GraphPad Software, San Diego, CA, USA). For comparison of sprouting index and axon tracing, a two-way analysis of variance (ANOVA) (followed by Student’s *t*-test with Bonferroni’s correction) was performed to determine differences between repair types and also repair position. Comparisons of axon disruption were made using a one-way ANOVA with Student’s *t*-test with Bonferroni’s correction. Differences were considered significant at *p *< 0.05.

## Results

**Fig. 3:**
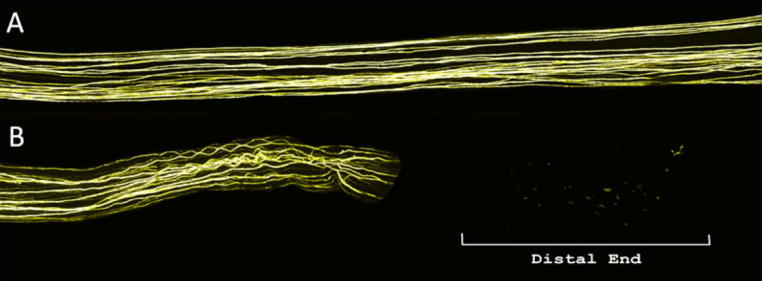
Effect of injury on axons distal to injury site. (A) Typical uninjured common fibular nerve section. (B) Transected and unrepaired common fibular nerve showing proximal and distal portions 2 weeks post injury; only minimal fluorescence is present in the distal portion.

All animals recovered well from the initial surgery with no signs of autotomy (self-mutilation of the denervated region) or infection. In all three animals where no repair was carried out, residual fluorescence in the distal portion of the nerve was minimal consisting of only small fluorescent flecks (Fig. 3B). This confirmed that any fluorescence in the graft and distal regions of repaired animals was from regenerating axons.

### Axon sprouting

**Fig. 4:**
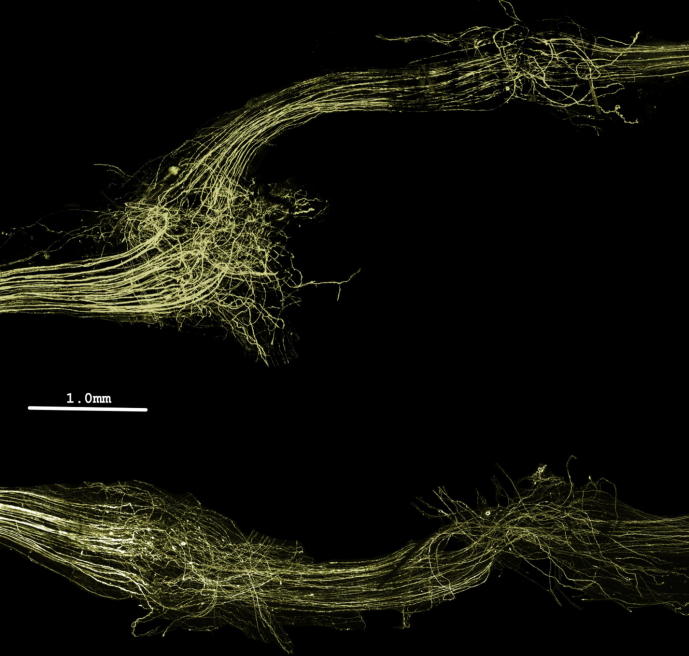
Images of nerve grafts. Typical vehicle- (top) and M6P-treated (bottom) nerve graft images. No obvious visual differences were noted between vehicle- and M6P-treated graft images prior to analysis.

Nerves from normal (uninjured) controls displayed clear individual nerve axons running in a near parallel course throughout the portion of nerve examined and no sprouting was observed (Fig. 3A). Following injury and repair significant sprouting was seen in both M6P- and vehicle-treated groups (Fig. 1, Fig. 2, Fig. 4). Sprouting was most apparent where the axons entered and exited the graft (Fig. 4).

**Fig. 5:**
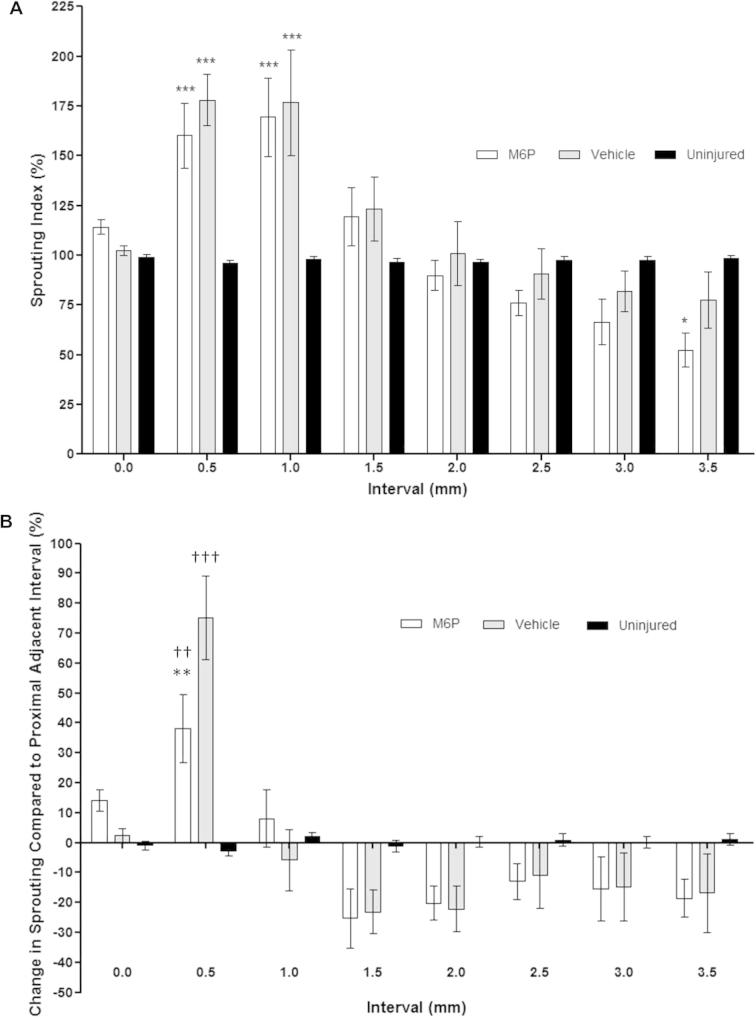
Quantification of sprouting. (A) Sprouting index values for each 0.5-mm interval. Differences in sprouting between repair groups were not significant; compared to uninjured controls sprouting in M6P- and vehicle-treated groups was significantly increased at the 0.5-mm and 1.0-mm intervals and in the M6P-treated group was significantly decreased at the 3.5-mm interval. Statistical test: two-way ANOVA with Student’s *t*-test with Bonferroni’s correction; ^∗^*p* < 0.05 compared to uninjured controls; ^∗∗∗^*p* < 0.001 compared to uninjured controls. (B) Changes in sprouting index levels compared to proximal adjacent site. Statistical test: two-way ANOVA with Student’s *t*-test with Bonferroni’s correction; ^∗∗^*p* < 0.01 compared to vehicle group; ^††^*p* < 0.01 compared to uninjured controls; ^†††^*p* < 0.001 compared to uninjured controls.

Quantification of sprouting revealed that M6P-treated grafts had a slightly lower sprouting index than the vehicle-treated group at the 0.5-mm and 1.0-mm intervals; this difference between treatment groups was maintained for the remaining intervals (Fig. 5A). However, these differences were not statistically significant (*p* > 0.05; two-way ANOVA with Student’s *t*-test with Bonferroni’s correction).

For both M6P- and vehicle-treated groups the maximum sprouting index values were observed at the 0.5-mm (160.2% [SEM = 16.3] for M6P and 178.1 [12.8] for vehicle) and 1.0-mm (169.4 [19.8] for M6P and 176.8 [26.6] for vehicle) intervals with values in both groups significantly higher (*p *< 0.001) than those of the uninjured group (99.1% [1.4] at 0.5 mm and 96.2 [1.1] at 1.0 mm). The lowest sprouting index for both repair groups was observed at the 3.5-mm (distal nerve stump) interval (52.5% [8.7] for M6P and 77.6% [14.4] for vehicle) with the sprouting index in the M6P-treated group being significantly lower (*p *< 0.05) than that for the uninjured group (98.5% [1.3]).

The maximum change in sprouting index compared to the previous level for both treatment groups occurred between the graft start and the 0.5-mm interval (Fig. 5B). The difference between repair groups was significant at this point (*p *< 0.01) with a greater increase seen in the vehicle-treated group (75.2% [SEM = 13.9] than the M6P-treated group (38.1% [11.3]). At this point the increase in sprouting index in both repair groups was significantly greater (*p *< 0.01 for M6P and *p *< 0.001 for vehicle) than that seen in uninjured controls (−2.8% [1.5]). The sprouting index values in both repair groups remained stable between the 0.5-mm and 1.0-mm intervals and then fell in both treatment groups between the 1.0-mm and 1.5-mm intervals. The sprouting index then continued to fall at a similar rate in both treatment groups between the remaining intervals (Fig. 5B).

### Axon tracing

**Fig. 6:**
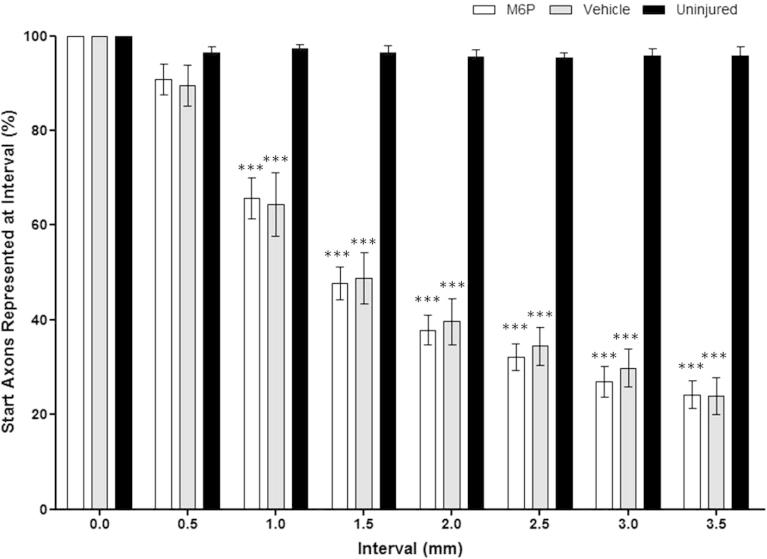
Proportion of axons from the start point represented at each subsequent site. No significant differences were observed between repair groups in the proportion of axons from the 0.0-mm site represented at each subsequent site. However, both repair groups had a significantly lower proportion of axons from the 0.0-mm interval compared to the uninjured group from the 1.0-mm interval to the 3.5-mm interval. Statistical test: two-way ANOVA with Student’s *t*-test with Bonferroni’s correction; ^∗∗∗^*p* < 0.001 compared to uninjured controls.

The proportion of individual axons from the start point (0.0 mm) represented at each interval along the graft declined at a similar rate in both M6P- and vehicle-treated grafts. No differences were noted between groups at each individual interval. In both groups approximately half of the axons at the start point failed to reach the 1.5-mm interval and three-quarters failed to reach the end of the graft (3.5-mm interval) (Fig. 6).

### Axon disruption

**Fig. 7:**
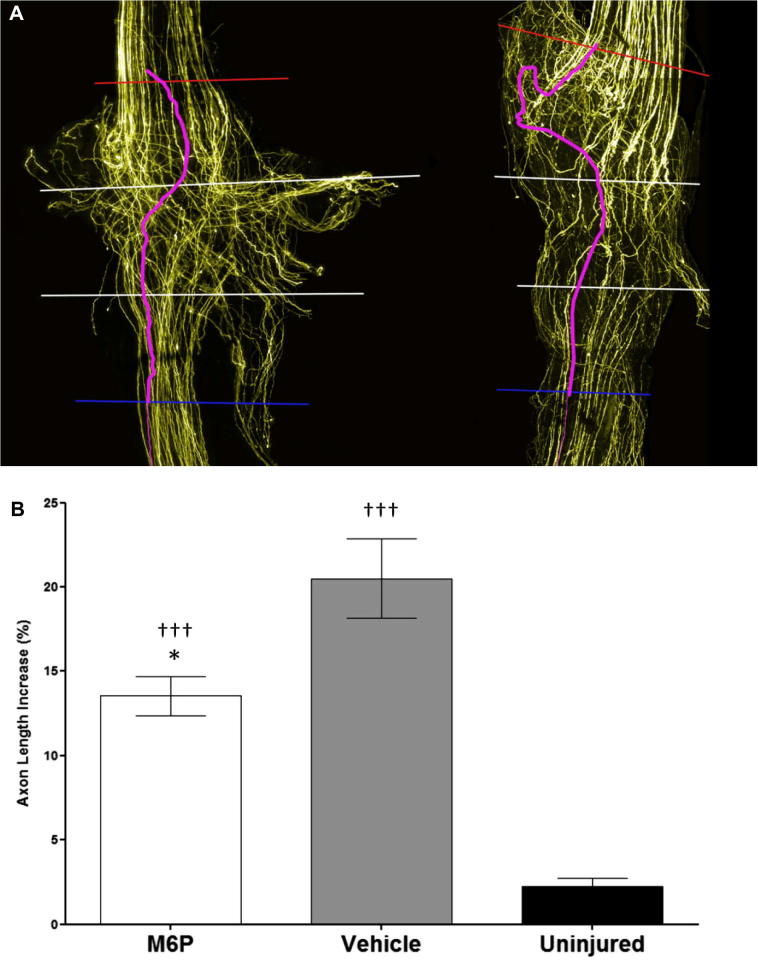
Comparison of axonal disruption. (A) Comparison of axon disruption in M6P- (left) and vehicle-treated repairs (right) across the initial 1.5 mm of the repair. An individual traced axon is used to indicate differences in axon length/disruption. (B) Average increase in axon length across the initial injury site. Axons in the M6P group were on average 13.55% (SEM = 1.17) longer than the distance between the graft start and 1.5-mm interval; axons in the vehicle group were 20.51% (2.36) longer. Statistical test: one-way ANOVA with Student’s *t*-test with Bonferroni’s correction; ^∗^*p *< 0.05 compared to vehicle group; ^†††^*p *< 0.001 compared to uninjured controls.

When crossing the proximal repair site the average increase in axon length between the graft start and the 1.5-mm interval was significantly lower (*p < *0.001) in the M6P-treated group (13.6% [SEM = 1.2]) than in the vehicle-treated group (20.5% [7.5]) (Fig. 7).

## Discussion

This study has allowed clear visualization of axon disruption following graft repair. It was clear following nerve imaging alone (Fig. 4) that a large amount of axon disruption occurred in both repair groups at the junction between the proximal and distal nerve endings and the graft. This was confirmed after analysis by the significant increase in sprouting index in both repair groups compared with the baseline for uninjured nerves at the 0.5-mm and 1.0-mm intervals (Fig. 5A). The additional repair site that axons must cross in graft repairs is known to be detrimental to nerve regeneration, with previous studies showing direct suturing under mild tension to be preferable to grafts – this despite the adverse effects on regeneration of tension at the repair site (Rodkey et al., 1980, Wong and Scott, 1991).

Regenerating axons will often branch into two or more sprouts following injury, with surplus axon branches degenerating with time (Bray and Aguayo, 1974, Toft et al., 1988). This branching increases the probability of an axon being able to navigate through to the distal nerve stump. Xu et al. (2008) found that axonal sprouting was significantly increased in a 20-mm-long nerve crush injury compared to a 2-mm-long nerve crush injury; this suggests that increased disruption within the nerve micro-environment, including that related to the development of intraneural scarring, can lead to increased axonal sprouting among the regenerating axons. Atkins et al. (2007) reported that a low dose of the anti-inflammatory cytokine IL-10 significantly reduced intra-neural scarring, and electrophysiological results indicated increased axon regeneration across the repair site. The axon counts distal to the repair for IL-10-treated nerves and saline-treated controls were, however, found to be similar; the reason for the similarity in axon numbers was thought to be related to additional sprouting in the saline-treated controls due to increased intraneural scarring (Atkins et al., 2007).

The application of M6P appears to have a significant impact on the amount of disruption that axons suffer at the early stage of regeneration into the graft. This is indicated by the significantly smaller increase in sprouting index between the graft start and the 0.5-mm interval (Fig. 5B) combined with significantly shorter average axon lengths over the initial 1.5-mm portion of the graft (Fig. 7). A previous study by Ngeow et al. (2011b) used measurements of compound action potentials to assess nerve regeneration and demonstrated that the application of M6P significantly improves nerve regeneration in the early stages of recovery, but that the improvement is not maintained later in the recovery process. The results of our study provide further evidence that M6P improves nerve regeneration and give an explanation for the findings of Ngeow et al., 2011a, Ngeow et al., 2011b. Our data indicate that the effect of M6P may be due to axons taking a more direct route across the repair site rather than regenerating at a faster pace or reducing the time taken for regeneration to be initiated.

The simplest explanation for the more direct route taken by axons across the repair site following M6P treatment is that collagen formation has been reduced by the inhibition of TGF-β activation. However, the studies by Ngeow et al., 2011a, Ngeow et al., 2011b did not find any significant reduction in collagen following M6P treatment, suggesting that the effects observed here may be due to differences in the alignment, density or thickness of the collagen fibers.

One possibility is that the application of M6P decreased the production rate of collagen for a short time following application, resulting in more organized initial collagen formation. Increased TGF-β1 is known to reduce the organization of collagen following tissue injury (Henderson et al., 2012) and increased collagen density is linked to reduced fibroblast movement through tissues, which causes decreased alignment of collagen (Dallon et al., 2001). So a reduction in collagen production during the early stages of peripheral nerve regeneration may lead to more preferentially arranged collagen fibers that help guide regenerating axons rather than obstructing them. Although previous studies did not find a significant reduction in overall collagen levels at 6 weeks post-injury (Ngeow et al., 2011a, Ngeow et al., 2011b), it is possible that the collagen production rate was significantly reduced at an earlier time point, allowing more organized collagen to form before the effects of M6P are countered and collagen production levels return to normal.

The lack of any notable difference between M6P- and vehicle-treated groups in terms of the proportion of axons regenerating from the graft start to the 3.5-mm interval does not clearly indicate an improvement in functional recovery. However, the proportion of axons reaching the distal nerve stump from the graft start is not the only concern when considering functional recovery. It has been shown that functional recovery is improved when axons regenerate faster through a graft (Danielsen et al., 1994) and that the denervated distal stump becomes progressively less capable of supporting axon regeneration over time (Sulaiman and Gordon, 2000). In addition, prolonged denervation may affect the ability of muscle fibers to receive regenerating axons (Miyamaru et al., 2008) and delayed repair has been shown to have a detrimental effect upon the recovery of muscle mass and motor function (Kobayashi et al., 1997). With the application of M6P allowing axons to take a shorter, and thereby faster, route through the main area of disruption there is the potential that the axons will reach the distal stump and their targets more quickly.

Within this study it was not possible to determine whether axons in M6P-treated grafts had actually regenerated further during the recovery period compared to those in vehicle-treated grafts. The reason for this is that leading axons had already regenerated past the point where the harvested nerve tissue was sectioned – just distal to the portion of nerve where the graft was inserted, the common fibular nerve passes around the lateral side of the knee joint and is difficult to access. As fluorescence persists in the distal stump for between 5 and 10 days (Beirowski et al., 2004) and regenerating tissues are extremely fragile after 7 days’ recovery (unpublished observations), reliable analysis after a shorter recovery period would be difficult.

## Conclusion

This study provides further evidence that M6P improves nerve regeneration in the early stages of nerve repair and indicates that the improvement is due to axons crossing the repair site in a quicker, more direct manner. How M6P effects this improvement is as yet unclear though it may be as a result of more favourably arranged collagen fibrils. This study also establishes the usefulness of our new methods of analysis using the *thy-1-YFP-H* mouse strain in tracing the path of axons across the injury site, and in visualizing and quantifying regeneration at the level of the individual axon.

## Role of the funding source

The MRC and Renovo plc provided studentship funding through an MRC Industrial CASE Studentship.

## Competing interests

There were no competing interests.

## Authorship

FMB and PPR conceived the study. FMB and CRC supervised the study. ARL supervised early pilot studies and MJF was the industrial supervisor. AJH prepared the animals and analyzed the data with assistance from CRC. FMB and AJH drafted the manuscript.
